# Ibero-American Consensus on Communication Skills for Nursing Degree students

**DOI:** 10.1590/1518-8345.5653.3523

**Published:** 2022-05-25

**Authors:** Ana María Pérez-Martín, Mercedes Gómez del Pulgar García Madrid, Roger Ruiz-Moral, Almudena Crespo-Cañizares, Cristina García de Leonardo Mena, Fernando Caballero-Martínez

**Affiliations:** 1 Universidad Francisco de Vitoria, Facultad de Ciencias de la Salud, Madrid, Espanha.; 2 Universidad Francisco de Vitoria, Facultad de Medicina, Madrid, Espanha.

**Keywords:** Nurse-Patient Relations, Health Communication, Graduate Nursing Education, Learning, Consensus, Delphi Technique, Relações Enfermeiro-Paciente, Comunicação em Saúde, Educação de Pós-Graduação em Enfermagem, Aprendizagem, Consenso, Técnica Delphi, Relaciones Enfermero-Paciente, Comunicación en Salud, Educación de Postgrado en Enfermería, Aprendizaje, Consenso, Técnica Delphi

## Abstract

**Objective:**

as a health care profession focused on caring for people, Nursing requires sound communication skills. Based on an international expert consensus, a proposal on learning outcomes in clinical communication for undergraduate Nursing education curricula in Spanish speaking countries is presented.

**Method:**

a steering committee, consisting of 5 nurses and experts in communication in health care sciences, drew up the first list of communication skills specific to the Nursing degree. Their proposal was reviewed and improved by a committee of 7 international scientific advisers. 70 experts from 14 countries were selected using a snowball sampling procedure and invited to participate in a distance modified Delphi consensus process in two survey rounds. Statistical analysis was carried out to establish the final consensus level for each item.

**Results:**

a questionnaire with 68 learning outcomes in clinical communications was submitted for panel assessment. In the first Delphi round, the panel reached a statistical consensus on all the items assessed. There was no need for a second round to reconcile positions.

**Conclusion:**

an academic proposal, approved by a high level of international consensus, is presented to guide and unify the learning outcomes on the clinical communication curriculum for undergraduate Nursing studies in Spanish speaking countries.

## Resumo

**Objetivo:**

como profissão orientada à atenção da saúde, focada no cuidado às pessoas, a Enfermagem precisa desenvolver habilidades de comunicação sólidas. Com base em um consenso internacional de especialistas, apresenta-se uma proposta sobre os resultados de aprendizagem na comunicação clínica para planos de estudos em cursos de Enfermagem em países hispanofalantes.

**Método:**

um comitê orientador, composto por 5 enfermeiros e especialistas em comunicação em ciências de atenção à saúde, elaborou a primeira lista de habilidades comunicacionais específicas para o curso de Enfermagem. Outro comitê de 7 assessores científicos internacionais revisou e melhorou a referida proposta. Foram selecionados 70 especialistas de 14 países pelo procedimento de amostragem “bola de neve”, sendo convidados para participar em um processo de consenso Delphi modificado a distância em duas rodadas de pesquisa. Foi realizada a análise estatística para estabelecer o nível de consenso final correspondente a cada item.

**Resultados:**

foi apresentado um questionário com 68 resultados de aprendizagem em comunicações clínicas para serem avaliados pelo painel. Na primeira rodada Delphi, o painel chegou a um consenso estatístico em todos os itens avaliados. Não foi necessária uma segunda rodada para conciliar posições.

**Conclusão:**

apresenta-se uma proposta acadêmica aprovada com um elevado nível de consenso internacional a fim de orientar e unificar os resultados de aprendizagem nos planos de estudo sobre comunicação clínica para cursos de Enfermagem em países hispanofalantes.

## Resumen

**Objetivo:**

en su carácter de profesión de atención de la salud enfocada en cuidar a las personas, la Enfermería requiere habilidades de comunicación sólidas. Sobre la base de un consenso internacional de expertos, se presenta una propuesta sobre resultados de aprendizaje en la comunicación clínica para planes de estudio de las carreras de grado de Enfermería en países de habla hispana.

**Método:**

un comité orientador, compuesto por 5 enfermeros y expertos en comunicación en ciencias de atención de la salud, elaboró la primera lista de habilidades de comunicación específicas para la carrera de Enfermería. Un comité de 7 asesores científicos internacionales revisó y mejoró dicha propuesta. Se seleccionó a 70 expertos de 14 países a través del procedimiento de muestreo “bola de nieve”, y se los invitó a participar en un proceso de consenso Delphi modificado a distancia en dos rondas de encuesta. Se realizó un análisis estadístico para establecer el nivel de consenso final correspondiente a cada ítem.

**Resultados:**

se presentó un cuestionario con 68 resultados de aprendizaje en comunicaciones clínicas para que lo evaluara el panel. En la primera ronda Delphi, el panel llegó a un consenso estadístico en todos los ítems evaluados. No fue necesaria una segunda ronda para conciliar posiciones.

**Conclusión:**

se presenta una propuesta académica, aprobada con un elevado nivel de consenso internacional, a fin de orientar y unificar los resultados de aprendizaje en los planes de estudio sobre comunicación clínica para carreras de grado de Enfermería en países de habla hispana.

**Highlights**

(1) The nurse-patient communication skills are a vital tool in their relationship.

(2) These skills influence the health care outcomes and the patient’s experience.

(3) There is heterogeneity between Nursing schools on communication teaching.

(4) A consensus from Spain/Latin-America experts intends to unify university objectives.

(5) A proposal of observable learning outcomes on nurse-patient communication is made.

## Introduction

The nurse-patient relationship is key to achieving the central purpose of Nursing care, that is, to help individuals and their families face the experiences of illness, suffering or disability effectively and acceptably, reducing their impact on the patient’s daily life. This type of interpersonal relationship is more complex and deeper than a simple accompaniment of instrumental actions usually associated with the Nursing work^1^ ^-^ ^3^. Nursing health care outcomes strongly depend on the nature of the nurse-patient relationship^4^ ^-^ ^5^. Research evidence indicates that a health professional’s ability to explain, listen and empathise^6^ ^-^ ^7^ is related to the patient’s capacity to follow through with medical recommendations, self-manage a chronic medical condition, and adopt preventive health behaviours. These communication skills (CS) can also improve the patients’ perception and satisfaction with the care they have received^8^. Furthermore, communication among health care team members influences the quality of the work relationships, job satisfaction and patient safety^9^. 

In recent years, the importance of CS in the education of future nurses and its inclusion in curricula has become increasingly evident^10^ ^-^ ^11^. In this sense, to achieve effective teaching/learning of CS in undergraduate studies in the health professions, among other strategies^12^, it is of utmost importance to identify the most appropriate curricular content for this education level and each health profession. With this in mind, in the medical professional field, several international documents^13^ ^-^ ^19^ have defined the contents and CS necessary to achieve efficient and ethical communication with patients. These proposals, with local nuances regarding their target audience and approach, have been useful to plan and develop training programs and appropriate evaluation strategies for health care workers in their different areas of influence. Some of these documents have an interprofessional health orientation with cross-cutting recommendations (e.g., the European consensus)^18^. Naturally, health professions share an important set of interpersonal skills. However, the practice of each particular profession involves specific aspects that encompass different types of interpersonal skills within the contexts where they are needed. For example, Nursing traditionally emphasises the importance of teamwork^20^ and its responsibility for aspects directly related to care^21^. In addition, theorists emphasise therapeutic communication to be of major importance in the Nursing practice and insist on the need to re-examine the Nursing care philosophy, moving beyond current limits to develop a more compassionate and humane approach^22^. The essence of such “person-centred” care requires nurses to be willing and able to establish a special type of relationship with their patients, one that is closer and more continuous than those experienced with other health care professionals^23^ ^-^ ^24^. 

The pre-existing curricular proposals on clinical communication^13^ ^-^ ^19^ were developed through expert consensus. In each case, the authors proposed a set of CS adapted to a specific framework (with its scientific evidence, cultural and professional determinants, and national or supranational legislation). In some cases^16^ ^-^ ^17^, these proposals were based on previous theoretical models of clinical communication, which helped to select and articulate the set of communication competences appropriate to each professional context. However, none of the previous competence proposals was specifically aimed at defining the CS competences of future nurses (the undergraduate students attending Nursing schools). Proposing an international, expert-supported framework on CS for undergraduate Nursing students would be useful to help each Nursing school select its educational objectives. In fact, there appears to be a need to clarify communication curricula in Nursing degree studies^25^ ^-^ ^26^. In Spain, for example, a recent study^27^ carried out in 110 Nursing schools (95.6% of the country’s total schools) explored the educational offer of Nursing communication education and the content covered in the degree curriculum. The study revealed that the teaching of these skills is both scarce and highly heterogeneous among the centres. This variance is not only present in the type or content of the skills required but also in the way and at what stage these CS are taught. In other Spanish-speaking American countries, Nursing schools are also in the process of integrating nurse-patient-family CS into their degree-level curricula. Having such a consensus would be of great use. Although there exist different proposals regarding the CS that should be acquired by Nursing professionals^28^ ^-^ ^32^, there are no similar statements on the teaching of CS within the Nursing degrees of these countries. 

The objective of this study has therefore been to develop a consensus on CS (defined as *learning outcomes* [LO]) specifically aimed at undergraduate Nursing education, bearing in mind the possible peculiarities of the Latin American cultural, social and educational context. Such a statement could help standardise the educative process about CS in Nursing schools and promote the application of more experiential and less theoretical learning and assessment methodologies in this field. 

## Method

This study uses a specific variant^33^ of the modified Delphi method^34^, designed to reach group consensus in a maximum of two rounds of a written survey with geographically dispersed panellists. Both are proposals derived from the traditional Delphi method^35^, to facilitate its applicability and improve its performance.

The steering committee (SC) of the project consisted of a multidisciplinary team made up by 5 members (3 university lecturers in Nursing, 1 expert in clinical communication and 1 expert in health care professions education), linked to *Universidad Francisco de Vitoria* (Madrid). This committee carried out the following tasks:

### Literature search

As an initial task, the SC asked the expert panel members for specific documents on clinical communication in Nursing education available in their respective countries (conceptual frameworks, curricular proposals, syllabus, educational reports and other related documents). An additional electronic literature search (PubMed 2000-2017, on Nursing Education, Clinical Communication and Professional Consensus) helped to find a key set of international articles on the topic (finally, 57 articles were selected and included as references in this study, after a peer-review process of each paper). This procedure was assisted by one expert on information research from a university library. After reviewing these materials, each Steering Committee member drafted and shared with the group his or her draft version of the learning outcomes (LOs) in clinical communication.

### A Nursing communication conceptual model

The group discussed the suitability of using a previously published conceptual proposal in clinical communication as a base for a communication model for Nursing^36^. The chosen framework is based on existing basic communication assumptions and main theories. This communication model has four key elements to consider during the encounter (interview) between health professionals and patients: 1. The people involved, i.e., the health professional and the patient, with their respective professional and family contexts. 2. The interactions established between the two agents during the communication process, both verbal and non-verbal, aimed, from the professional’s perspective, at developing the tasks pursued in the relationship (connecting, identifying, understanding, agreeing, helping). 3. The clinical contexts in which communication occurs and which may condition it (specific health problems, emotional or sensitive situations, age or socio-cultural factors, health promotion, etc.). 4. The communication channels, as the medium used to transmit the message and key communication “intermediaries” (face-to-face, telephone, written or electronic). The model also considers other communication needs of the health professional in addition to those of the patients (communication with the patient’s family, with other health professionals or with the health authority).

### Development of the survey questionnaire

Based on this model^36^ and the official framework document on the contents of the teaching guides for the Nursing degree in Spain^37^, our multidisciplinary SC completed the contents and adapted the structure of its draft on LOs in communication written in the previous phase. The final format of this Nursing-specific questionnaire shares its main thematic blocks with the list of LOs for medical communication^19^, previously developed from the same conceptual framework, by a partly common research team, with a similar methodology and in the same Latin American geographical and cultural context. 

This second draft was passed on for critical review by 7 international scientific advisers which were not involved with its development, constituted as the Advisory Board overseeing the project. This committee received the questionnaire and a dossier with details about the conceptual model, together with a report on the technical bibliography consulted. Suggestions for possible improvements to the questionnaire (extension, reduction or amendment of items) were collected and shared electronically. Taking into account all suggestions and observations, the SC created a final draft on Nursing degree communication skills (LOs), which was again unanimously approved by the International Advisory Board. This final version of the questionnaire used in the survey rounds included 68 possible communication LOs considered appropriate for Nursing and grouped into the six categories (skills areas) related to the conceptual framework on clinical communication chosen for the project^36^: communication with the patient, communication with the patient’s family, intrapersonal communication (self-perception), inter-professional communication, communication through different routes/channels and communication in special situations. During the development process, an expert in Applied Pedagogy was available on an ongoing basis to ensure that the learning outcomes were properly written in their correct format according to Bloom’s taxonomy^38^.

### Selection of the panellists

Subsequently, panellist candidates were selected through the snowball sample technique^39^. Recruitment started from the network of professional contacts of the local SC and the international Scientific Committee, as well as other potential experts identified during the literature search, according to the inclusion criteria proposed. Based on these first elements, a cascade selection process was triggered, with no limitations throughout Latin America. All professionals who received more than one nomination for expert recognition by their peers were invited to participate. 

**Figure 1:**
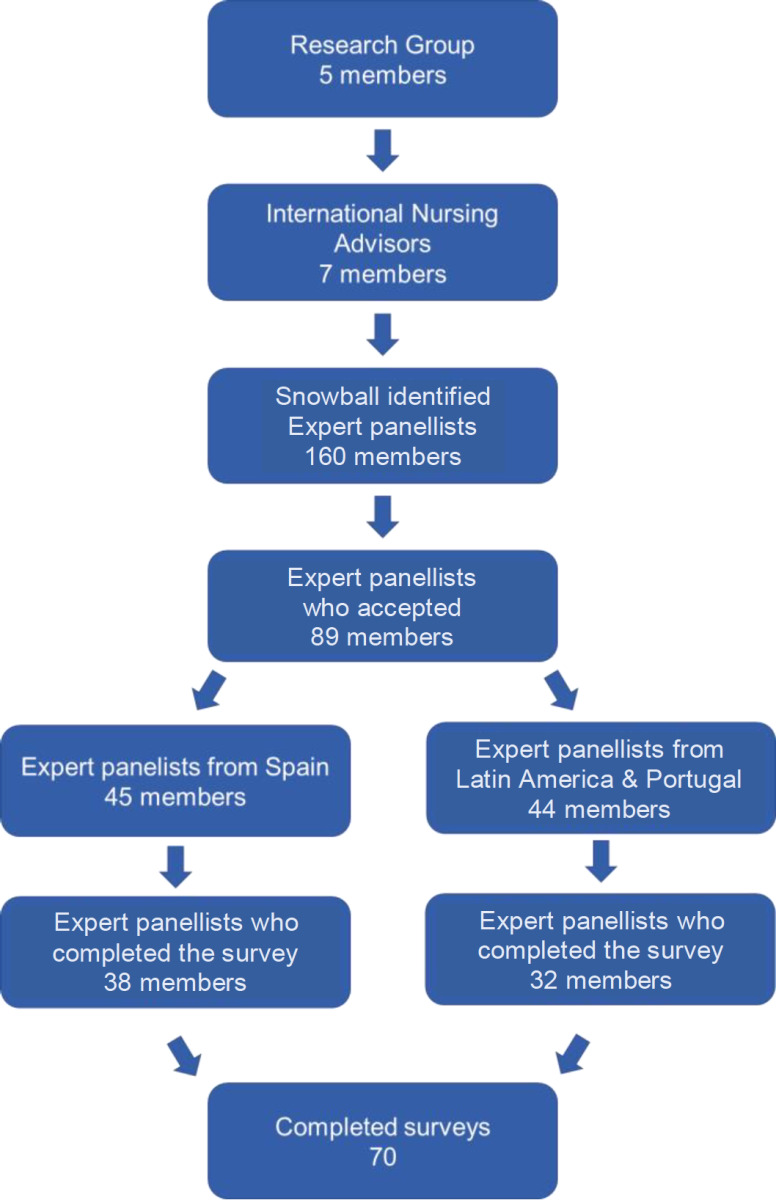
Participants in the successive phases of the study

To assemble an international panel of Nursing experts^40^ with diverse and complementary profiles, during the nomination process, candidates were sought with one or more of the following eligibility criteria: recognised leadership in clinical communication; experience as an educator in a Nursing school; position of institutional responsibility (educational, care, scientific or associative); wide range of health care experience (public/private, rural/urban). The procedure for acceptance of candidates was supervised by the Scientific Committee. Finally, 160 experts from 14 countries were identified (Spain, Portugal, Argentina, Chile, Costa Rica, Ecuador, El Salvador, Guatemala, Honduras, Mexico, Nicaragua, Panama, Paraguay and Uruguay). Eighty-nine of them preliminarily accepted the invitation to participate in the panel of experts under the established conditions (anonymity and free of charge) (Figure 1). 

The final panel of subjects who participated in full consisted of 70 experts, 38 of whom were Spaniards and 32 from different Latin American countries and Portugal, of which 80% were women and 20% were men. 54% of the panellists enjoyed PhD academic status. Regarding their professional profile, 82.9% professed a primary dedication to Nursing teaching, followed by health management professionals (30.0%), research (22.9%) and health care practice (28.5%), with a balanced distribution between hospital specialities and primary care services. The experience in their respective jobs was diverse, being especially prolonged (>10 years) among the panellists with a dedication to health care (49.3%) and management (42.9%). No information was requested regarding the respondents’ ethnic profiles. Their work location was essentially urban, as is expected in a group of people mainly devoted to teaching. 66% of the panellists’ main work was in public institutions, while the remaining smaller group was linked to private institutions or organisations.

All participants previously declared absence of conflict of interest and confirmed they possessed sufficient motivation and time to collaborate in the project. The carry-over process was requested on two consecutive occasions from the first generation of candidates, the process being repeated in each new country in which an expert was identified.

### The survey, evaluation of the items by the panel and consensus criteria

The variant of the Delphi method used in the study^30^ makes it possible to offer, as required for each item under analysis, up to two consecutive rounds of an electronic written survey to approximate the experts’ positions and reach a consensus. To express their opinion on each item under discussion, the panellists used a 9-point Likert ordinal scale, according to the format developed at the UCLA-Rand Corporation for the method of assessing the appropriate use of health care technology^41^. The response categories on this scale were grouped into three regions (1-3 = “disagree”; 4-6 = “neither agree nor disagree”; and 7-9 = “agree”). The questionnaire offered the possibility for the participants to include free comments. Non-scored items were treated as lost cases for statistical purposes.

To analyse the group opinion and the type of consensus reached, the position of the median of the group scores and the level of agreement reached by the respondents were used according to the following criteria^41^: an item was considered consensual when there was opinion “concordance” in the panel; that is, when the experts who scored outside the region of three points containing the median ([1-3], [4-6], [7-9]) were less than one-third of the respondents. In addition, the value of the median score determined the group consensus reached as “majority*”* disagreement with the item if the median was ≤ 3, or “majority*”* agreement with the item if the median was ≥ 7. Items with cases in region (4-6) were considered “dubious”. Panel criteria “discordance” was considered when the scores of one-third or more of the panellists were in region (1-3) and of another one-third or more in region (7-9). 

After the first survey round, the experts received a report with the distribution of the group’s opinions and a transcript of the comments collected from their peers. This feedback is intended to allow the panellists, if necessary, to reconsider their opinions on controversial issues using a new survey round. The free comments collected in the first round also allow the scientific committee to assess the need for improvement or clarification in the wording of an item. These comments were analysed qualitatively and a list of categories was derived inductively. In our study, the high level of agreement obtained in the first round and the panellists’ opinions collected allowed us to confirm consensus and close the process without the need for further rounds. 

This research project was favourably assessed by the Research Ethics Committee of *Universidad Francisco de Vitoria* (Madrid), with protocol number 3/2020.

## Results

Of the 89 experts who agreed to participate and served on the panel of experts, 70 completed the study. The national origins of the panellists were Spain (38), Portugal (2), Mexico (6), Guatemala (2), Honduras (2), El Salvador (2), Nicaragua (1), Costa Rica (2), Panama (3), Ecuador (3), Chile (1), Paraguay (2), Uruguay (2) and Argentina (4). Concerning the 68 learning outcomes proposed for evaluation in the questionnaire, this panel reached a sufficient agreement degree in the first survey round according to the pre-established consensus criteria (Table 1).

**Table 1:** Overall results of the study: confirmatory indicators of the group consensus reached in the 68 communicational learning outcomes analysed

A) COMMUNICATION WITH THE PATIENT				
A.1. General aspects of the Clinical Interview with patients.Recognise the value of the clinical interview for the preparation of the care plan, knowing, integrating and structuring its different components.Learning outcomes:	x̄^*^	M^†^	IQR^‡^	% out M^§^
Describe the different communication skills (verbal and non-verbal), or relationship skills, needed to prepare history of care needs prepared by nurses.	8.01	9	2	9.46
Recognise the mechanisms through which clinical communication influences health care outcomes (Demonstrate kindness, empathy, interest, active listening, satisfaction, perceived self-efficacy, trust, increased adherence).	8.55	9	1	1.35
Carry out a personalised clinical interview integrating the contents of the Nursing context with the communication and relationship skills.	8.7	9	0.5	1.35
A.2. Tasks and Skills to communicate with patients.Learning outcomes:	x̄^*^	M^†^	IQR^‡^	% out M^§^
A.2.1. Establish and maintain a therapeutic relationship (Connect) (The student establishes and maintains a therapeutic relationship through a patient-centred approach)				
Establish a nurse-patient relationship in which the patient feels comfortable and listened to regarding his/her needs.	8.69	9	1	0
Perceive the patient’s non-verbal language (mimic, kinaesthetic, proxemic, and tactile) and respond appropriately to the context.	8.53	9	1	1.35
Use medical history records (paper/computerised) in communication with the patient in a way that reduces or avoids interference.	7.69	8	2	13.51
Apply social skills to receive patients which encourage the establishment of an effective relationship (greet, call the patient by their name, make them feel comfortable, smile...).	8.78	9	0	0
Apply social skills to say goodbye to patients, which encourages maintaining an effective relationship (say goodbye cordially, accompany, thank...).	8.72	9	0	1.35
Recognise the patient’s emotions in different contexts, difficult situations and communication challenges (crying, strong emotions, interruptions, aggressions, anger, anxiety, sensitive or embarrassing issues, cognitive difficulties, bad news, first encounter...).	8.59	9	1	0
Respond emphatically (explore the origin of emotions, understand them and communicate understanding) to the patient’s emotions in difficult situations and communication challenges.	8.55	9	1	0
Establish a relationship with the patient based on respect and consideration of their rights, autonomy, beliefs, values and individuality as a human being.	8.72	9	0	2.7
Use a sense of humour in the relationship with the patient (in situations that require the surroundings to be relaxed, for the approach...).	7.85	8	2	13.51
A.2. Tasks and Skills to communicate. Learning outcomes with patients:	x̄^*^	M^†^	IQR^‡^	% out M^§^
A.2.2. Exchange information and understand it.				
A.2.2.1. Obtain the information (The student collects the relevant information for the proper development of the Nursing work).				
Recognise the advantages and disadvantages of different communication skills (open/closed questions, facilitation...) in order to obtain information.	8.19	8.5	1	1.35
Use verbal and non-verbal techniques of active listening (paraphrasing, facilitating speech, showing low reactivity, capturing clues, summarising...).	8.54	9	1	0
Summarise the information obtained as a form of verification to the patient.	8.41	9	1	4.05
Establish an adequate accompaniment of the physical examination (asking for permission, explaining what you propose to do and why, sharing the findings with the patient...).	8.69	9	0	1.35
A.2.2.2. Offer the information (The student offers the information in a clear and personalised way, which the patient needs to understand, accept, implement the plan of care).				
Estimate the patient’s level of knowledge about their problem and how much they wish to know in order to deliver the amount of information they really need and can be given.	8.38	9	1	4.05
Adequately communicate risks and possible discomforts to the patient, during Nursing care.	8.61	9	1	1.35
Properly use information aids (written, graphical, etc.) and instructions to supplement verbal information when necessary.	8.51	9	1	0
Adapt communication to the patient’s level of comprehension and language, avoiding technical terms.	8.61	9	0	4.05
Provide information to the patient in a timely manner (appropriate circumstance).	8.47	9	1	4.05
Explain to the patient the benefits, risks and expected outcomes of interventions derived from the Nursing care process.	8.72	9	0	0
Check that the patient has understood the information provided, facilitating the expression of doubts.	8.74	9	0	1.35
Refer the patient to the most appropriate professional when the nurse’s level of competence in information requirements is exceeded.	8.53	9	0	5.41
Transmit information related to Nursing care, in a manner adapted to the patient’s degree of tolerance and needs.	8.54	9	1	1.35
Share, with the patient’s consent, the information with third parties (colleagues, family and others...), when both consider it necessary and/or if the patient requests it.	8.26	9	1	6.76
A.2.3. Agree and Assist the patient in carrying out what has been agreed on for the care plan (promote the patient’s participation, taking into consideration the patient’s capabilities to develop and implement the care plan proposed).				
Identify and assume their Nursing role in the decision-making process of each patient’s individualised plan.	8.23	9	1	6.76
Explore the patient’s disposition and capacity (information, autonomy, trust, responsibility, psychological traits...) to facilitate their involvement in the care process.	8.53	9	1	1.35
Reach agreements with the patient using negotiation skills.	8.19	9	1	8.11
Clarify, when appropriate, how and when the agreed upon decisions should be taken (abandoning toxic habits, change of diet, etc.).	8.36	9	1	2.7
Share the range of possible consequences of a decision with the patient.	8.14	9	1	9.46
Offer the patient the option of involving third parties (colleagues, relatives) in the decision-making process.	7.71	8	2	15.07
**B) COMMUNICATION WITH THE PATIENT’S FAMILY**				
B.1. The patient’s family context (The student recognises and evaluates the role of the family in the patient’s care and establishes effective communication with the family for the patient’s benefit).Learning outcomes:	x̄^*^	M^†^	IQR^‡^	% out M^§^
Request and evaluate relevant information from other family members and caregivers of the patient, if this is necessary and available.	8.29	9	1	5.56
Assist the family in the process of caring for minors or disabled patients (dementia, coma patients, incapacitating mental problems...).	8.51	9	1	2.74
Recognise specific communication challenges with family members (confidentiality, secrecy, the sick companion, the need to accompany...).	8.34	9	1	5.48
**C) INTRAPERSONAL COMMUNICATION (SELF-PERCEPTION)**				
C.1. The nurse as a person (self-knowledge, self-reflection, self-criticism and self-care) (The student usually reflects on his/her behaviour and the way in which he/she communicates, developing and improving his/her self-knowledge, self-reflection, self-criticism, self-care).Learning outcomes:	x̄^*^	M^†^	IQR^‡^	% out M^§^
Distinguish the main sources of errors, related to communication failures that may jeopardize patient safety (poor information or assessment of patient needs, inadequate understanding...)	8.48	9	1	0
Recognise the cognitive biases (deficiencies or lack of knowledge updating) that hinder development of the Nursing work.	8.34	9	1	5.48
Recognise negative emotions (insecurity, antipathy, rejection...) that can make Nursing difficult, to distance oneself from them and create empathy.	8.37	9	1	4.11
Use strategies to reduce stress and overload (relaxation, reflection groups, Balint groups, supervision and support...).	8.26	9	1	10.96
Control one’s own emotional reactions and work efficiently, even in difficult situations (patient with high degree of suffering, demanding patient...).	8.47	9	1	0
Develop self-knowledge strategies required for the recognition of own biases, through the use of specific techniques (reflexive questions, observation with perspective, full presence [mindfulness], suspension of judgement, non-judgmental attitude, etc.).	8.36	9	1	2.74
**D) INTER-INTRA-PROFESSIONAL COMMUNICATION**				
D.1. The nurse’s professional context: Inter- and intra-professional communication.(The student communicates efficiently with professionals who are part of his/her team or outside it).Learning outcomes:	x̄^*^	M^†^	IQR^‡^	% out M^§^
Facilitate the flow of information from the opinions in the team and willingly allow and accept that team members give diverse opinions.	8.41	9	1	5.41
Provide feedback to team members appropriately (first-person comments, highlight the positive first, do not judge).	8.47	9	1	1.35
Contribute effectively to continuity of care in reference/referral and return of patients between different care levels (primary, specialised).	8.43	9	1	1.35
Carry out clinical or scientific presentations in public effectively.	8.46	9	1	2.7
Give clear and precise instructions to team members.	8.47	9	1	4.5
Contribute to creating a positive work atmosphere through the use of collaborative, non-hierarchical strategies.	8.43	9	1	2.7
Maintain confidentiality about decisions made in the team.	8.64	9	0	2.7
Respect individuality, the subjective perception of team members and the mastery (expertise) of different health care professionals by accepting differences constructively.	8.61	9	1	2.7
Be assertive with the rest of the team members.	8.61	9	1	2.7
**E) COMMUNICATION BY DIFFERENT MEANS**				
E.1. Communication Channels (The student efficiently uses different ways of communicating).Learning outcomes:	x̄*	M^†^	IQR^‡^	% out M^§^
E.1.1. Direct communication (face-to-face).				
Identify whether there is a discrepancy between the verbal and non-verbal components of communication.	8.38	9	1	1.35
Properly use proxemic communication (physical distance of communication).	8.26	9	1	6.76
E.1.2. Written communication.				
Recognise the formats and supports of clinical histories and the documents usually used for written communication with patients and between professionals (discharge reports, referral, request for tests...).	8.61	9	1	2.7
E.1.3. Computer or electronic communication.				
Manage information technologies (office automation, typing, e-mails, WhatsApp, web2.0...) in health care aspects, guaranteeing confidentiality.	8.35	9	1	8.11
E.1.4. Telephone communication.				
Recognise the uses and limitations of telephone communication with patients.	8.2	9	1	8.11
Communicate by telephone with patients attending to the specific demands and communication adaptations that this medium requires.	7.54	8	2	16.22
**F) COMMUNICATION IN SPECIAL SITUATIONS**				
F.1. Specific communication contexts (The student applies and adapts the core communication skills to specific clinical situations and uses specific skills that each situation may require).Learning outcomes:	x̄^*^	M^†^	IQR^‡^	% out M^§^
F.1.1. Sensitive situations.				
Recognise delicate situations that represent communication challenges (such as giving bad news, dealing with end-of-life issues, mourning situations, sexual history, gender violence, child abuse, HIV infection, explaining situations of clinical uncertainty...).	8.49	9	1	5.41
Address delicate situations sensitively and constructively by applying specific strategies and skills that each situation may require, such as empathy and sensitivity.	8.77	9	0	0
F.1.2. Management of emotions.				
Recognise situations of emotional tension in consultations (such as stress, fear, anger, aggressiveness, denial, collusion, shame...).	8.58	9	1	2.7
Address situations of tension in a sensitive and constructive manner by applying specific strategies and skills that each situation may require.	8.41	9	1	5.41
F.1.3. Cultural and social diversity. The student will be able to...				
Recognise the patients’ cultural and social diversity (ethnicity, nationality, socio-economic status, language, religion, gender, values, sexuality...) and the communication difficulties that this entails.	8.5	9	1	5.41
Address the cultural and social diversity of the patient and family by applying specific strategies and skills that each may require.	8.45	9	1	4.05
F.1.4. Health promotion and behavioural change.				
Identify the patient’s accessibility to adopt healthy behaviours.	8.43	9	1	5.41
Apply motivational and effective communication strategies to modify individual behaviours.	8.43	9	1	4.05
Promote the implementation of healthy behaviours through individual and group communication techniques.	8.43	9	1	5.41
Use group communication techniques to promote health and encourage the modification of healthy behaviours.	8.41	9	1	4.05
F.1.5. Specific clinical contexts.				
Adapt communication skills and strategies to the different specific psychiatric contexts, patients with dementia, with sensory problems: auditory, visual, verbal expression.	8.62	9	1	1.35
F.1.6. Patients of different ages.				
Adapt communication skills and strategies to different patients belonging to different age groups (children and parents, adolescents, elderly).	8.66	9	1	1.35

* x̄* = Mean score; ^†^M = Median score; ^‡^IQR = Interquartile range; ^§^% out M = Percentage of experts located outside the three-point area in which the median score is included

For each appraised item, the table details the statistics which establish the degree of consensus reached: the median score and the arithmetic mean of the panel scores, the proportion of experts who voted outside the three-point region that includes the median (against the majority of the group) and the interquartile range as a measure of panel variability. In all cases, the medians were located in the region of 7-9 points (agreement) and the proportion of panellists in the inverse region (1-3) did not exceed one-third of the group. The panel of experts reached a sufficient consensus in the first round of the procedure for all the learning outcomes proposed. In the absence of express suggestions in the questionnaires, it was considered unnecessary to carry out a second survey round, given that there were no outstanding issues to be clarified or resolved after the first iteration (Table 1).

The free comments and/or suggestions made by the panellists after the first round were analysed by pairs formed among the researchers, none of which were cause for the modification of the initial proposal of the researchers (list of learning outcomes) in any way. Most of the comments expressed their appreciation or highlighted the importance of LOs to be included in the undergraduate Nursing curricula. Other comments were related to (i) Practical implementation of LOs in undergraduate teaching, (ii) Clarification of the meaning of LOs, avoiding cultural misunderstandings, (iii) Implications for teaching and assessment methods.

The mean value of the panellists’ scores for each of the 68 assessed learning outcomes was used, for comparative purposes, to rank the items according to the degree of approval received by the expert panel. Under this criterion, a group of 17 skills were selected, which reached the highest degree of panel endorsement (whose mean score was placed in the upper quartile of the distribution of averages, that is, with a mean score higher than the 75 percentile value = 8.31). These learning outcomes can be considered a priority for the experts consulted, composing a “skills core*”* of special interest (Table 2).

**Table 2:** Core communication skills (items that obtained mean scores higher than percentile 75 of the panel distribution)

No.	Agreed items with mean scores > 8.306 (> percentile 75)	Mean
P2	Recognise the mechanisms through which clinical communication influences health care outcomes (Demonstrate kindness, empathy, interest, active listening, satisfaction, self-efficacy, self-perception, trust, increased adherence).	8.56
P3	Carry out a personalised clinical interview integrating the contents of the Nursing context with the communication and relationship skills.	8.60
P4	Establish a nurse-patient relationship in which the patient feels comfortable and listened to regarding his/her needs.	8.59
P5	Perceive the patient’s non-verbal language (mimic, kinaesthetic, proxemic, and tactile) and respond appropriately to the context.	8.43
P7	Apply social skills to receive patients which encourage the establishment of an effective relationship (greet, call the patient by their name, make them feel comfortable, smile...).	8.69
P8	Apply social skills to say goodbye to patients, which encourages an effective relationship to be maintained (say goodbye cordially, accompany, thank...).	8.63
P9	Recognise the patient’s emotions in different contexts, difficult situations and communication challenges (crying, strong emotions, interruptions, aggressions, anger, anxiety, sensitive or embarrassing issues, cognitive difficulties, bad news, first encounter...).	8.41
P10	Respond emphatically (explore the origin of emotions, understand them and communicate understanding) to the patient’s emotions in difficult situations and communication challenges.	8.45
P11	Establish a relationship with the patient based on respect and consideration of their rights, autonomy, beliefs, values and individuality as a human being.	8.63
P14	Use verbal and non-verbal techniques of active listening (paraphrasing, facilitating speech, showing low reactivity, capturing clues, summarising...).	8.33
P16	Establish an adequate accompaniment of the physical examination (asking for permission, explaining what you propose to do and why, sharing the findings with the patient...).	8.49
P18	Adequately communicate risks and discomforts to the patient, during Nursing care.	8.40
P20	Adapt communication to the patient’s level of comprehension and language, avoiding technical terms.	8.40
P22	Explain to the patient the benefits, risks and expected outcomes of interventions derived from the Nursing care process.	8.50
P23	Check that the patient has understood the information provided, facilitating the expression of doubts.	8.54
P25	Transmit information related to Nursing care, in a manner adapted to the patient’s degree of tolerance and needs.	8.34
P28	Explore the patient’s disposition and capacity (information, autonomy, trust, responsibility, psychological traits...) to facilitate their involvement in the care process.	8.34

Overall, and related to the different thematic sections of the survey, the items belonging to the “nurse-patient communication” and “communication with the patient’s family” blocks reached the highest degree of panel approval (highest mean score).

## Discussion

The Spanish-American Consensus on Communication for Nursing (in Spanish, *Consenso Hispanoamericano en Comunicación para Enfermería*, or CHCE) represents a consensual statement on educational outcomes in health care communication for Nursing degree studies, something which never before existed among Spanish-speaking countries. It expands and reinforces some of the proposals that have been made on communicative competences for the Nursing profession within the framework of the profession’s general competences^10^ ^,^ ^28^ ^-^ ^32^. At the end of this process, there were 70 experts from Spain and different Ibero-American countries who developed this basic communication curriculum intending to serve as a guide to help establish the communicative learning outcomes that Nursing training in higher education may provide. 

The size of this panel is larger than that used in another consensus. When, as in our case, the hypothetical population of experts available to be recruited is large and international in scope, the ideal size of the group surveyed should be considered. Although a formal sample size calculation has never been considered necessary, in our case it seems reasonable to oversize the panel to make it more representative. Moreover, experimental evidence has shown the direct relationship between the size of the expert panel and the precision of the group estimate obtained (the expert forecast error tends to decrease exponentially as the panel size increases)^33^.

Looking at its content and methodology, this consensus is aligned with the main statements on the teaching of CS within other health professions^13^ ^-^ ^19^. It represents a concretion for the teaching of Nursing similar to that of the LAPS-CCC (Latin American, Portuguese and Spanish consensus on a Core Communication Curriculum, for undergraduate medical education), for the degree of Medicine in the Ibero-American cultural context^19^, as both proposals have used the same conceptual model on clinical communication as a framework^36^, together with the same methodological consensus strategy.

Today, the Nursing professional’s ability to be a good communicator is considered essential: These CS are therefore found in almost all proposals or statements on the generic competences that we have reviewed for the practice of this health care profession^10^ ^,^ ^28^ ^-^ ^32^. Some of these, such as the International Nurse Council^29^, the Professional Nursing Standards of ANA^31^, or the 30 areas on skills identified as necessary to practice Nursing in Australia^30^, offer a varied and wide range of communicative skills or competences (this ranges between approximately 45 and 67). The current proposal is in line with these statements and proposals in terms of the number of proposed communicative outcomes (68) as well as in the nature and content of the majority of them. Despite this, CHCE offers several particularities that we believe provide additional value as a whole. Generally speaking, and for different reasons, the implementation of competing proposals in the teaching of the degree proves to be difficult^26^ ^,^ ^42^. In many cases, this leads to lack of training in some of them, which is also true for those of a communicational nature^43^ ^-^ ^47^. Some of the peculiarities of CHCE may be particularly useful in encouraging and facilitating the incorporation or improvement of the teaching of these skills, both in the cultural environment in which it has been carried out and also in others that may or may not share some of their cultural characteristics. In the first instance, this proposal, like others in other health professions^13^ ^-^ ^19^, includes only skills or competences exclusively of a communicative nature, which can help more when considering the set of communicative aspects most relevant to the teaching of Nursing. In addition, a list is provided derived from a conceptual framework^36^, which coherently includes the people involved (nurse and patient) and some of their main relational determinants, their interactions in the context of a generic Nursing interview and also in other specific health contexts and communication channels. Other proposals, such as the UK communication curriculum content for medical degree education^16^, are framed in what is called the “*communication curriculum wheel’’*. This “wheel” also aims at facilitating a better understanding of the importance of contemplating a particular set of skills as well as other elements of communicative content in higher education. In most of the Nursing competence proposals reviewed^10^ ^,^ ^28^ ^-^ ^32^, the communicative competences are offered together with others of a different nature and usually distributed in different domains and subdomains, some of them very generic such as “Professional practice”, “Provision and coordination of care”^32^, “Accountability” and “Ethical practice”^29^. Doing so through a conceptual framework such as the one that underpins CHCE perhaps offers greater coherence to the different communicative domains where the 68 learning outcomes proposed are found. Consequently, this may facilitate the consideration and suitability of the different skills throughout the different curricula. Furthermore, an important effort has been made to offer these outcomes as observable behaviours. With this in mind, they have been written as “Learning Outcomes” (LOs) and following the taxonomies of Bloom’s educational objectives^38^. We therefore try to avoid debate that identifies them as “competences”, as this often leads to greater difficulties when setting out the LOs for teaching in a practical manner^48^. Most of these LOs are based more on behaviour and attitude than on cognitive activity. Although it might well involve several practical challenges for some institutions, incorporating them into a Nursing curriculum will also prove useful as a guide for setting out teaching and evaluation methodologies. It seems logical that, because of the nature of these LOs, for a student to incorporate and apply them, the educational institution must prioritise experiential teaching methods^15^ ^,^ ^49^ ^-^ ^50^. Such methods must contemplate repeated exposure to a variety of clinical situations in which students can be observed, receive structured feedback, have enough time to reflect on what has been learned and then practise under simulated conditions. As a general rule, their teaching will require organising not in isolated courses, but throughout the curriculum and be taught by adequately trained teachers^15^ ^,^ ^51^ ^-^ ^52^.

All of the above represents added value to this LO proposal, one that may be of great use to many Nursing schools in the design and modification of their programs more efficiently and effectively^27^. However, it is important to bear in mind that many of these objectives require the student to understand subtle aspects of the hidden curriculum and develop intuitive thinking^53^. This can only be accomplished slowly and with frequent and reflective practice^53^ ^-^ ^54^. Nursing students are young, mostly inexperienced, and often have difficulty incorporating these types of skills^44^ ^,^ ^55^. This learning is by no means easy and, therefore, some adaptation and customization of the experiential educational strategies will always be required throughout their undergraduate program^26^ ^,^ ^44^ ^-^ ^45^.

General acceptance of these LOs by the communication experts participating in the consensus was very high, especially for 17 of them, which were therefore considered to be the core group. In the “patient-nurse” communicative domain, these LOs highlight essential communicative aspects related to establishing and maintaining a therapeutic relationship (6 LOs), attending and responding to emotions (2 LOs), obtaining information (1 LO) and above all, providing it in an understandable and adaptable manner (6 LOs). 

The nature of these LOs represents the central character that experts seem to give to “person-centred care” in the field of the Nursing clinical practice and serve to form a basic and interesting “core curriculum” or starting point for teaching communication in Nursing schools. In addition, this reference document could help clarify other aspects of interest, such as where and how different skills should be trained. It could also serve as a guide to identify possible academic subject gaps in a given curriculum. On the other hand, this proposal can also be useful to facilitate the coordination of student exchanges between schools and health care providers from different countries. For example, the European Higher Education Area encourages the use of common competences in different countries to facilitate comparison of curricula and mobility of students between European Nursing schools^56^. 

## Limitations

Both the phases that are before the Delphi process and the survey process are subjected to various types of bias. The consensus reflects the opinion of the 82 people (of the steering committee, scientific advisory committee and panellists) who participated in the previous phases of literature review and selection, acceptance of the conceptual model and selection and pre-adaptation of the 68 items from LAPS-CCC that were finally agreed upon by the experts participating in the Delphi study. Another group of participants could have reached slightly different conclusions. However, our consensus document is not alone in this criticism. Consensus methods in general have been criticised for their limited scientific nature^57^ ^-^ ^58^. However, the Delphi method has been widely studied and used in the health sciences, particularly in Nursing^59^.

It has been questioned whether reaching consensus is a scientific method or simply a way of structuring group communication. However, it should be borne in mind that CHCE is a proposal for the development of LOs and that objectives of this type always require value judgements. The most important aspects of this entire process include the identification and selection of experts, leaders and the scientific committee of the study. CHCE comprised a wide variety of participants from different fields of clinical Nursing and Nursing education. Their geographical origin was quite diverse, although not proportionate (for example, the American countries had relatively few representatives while the Spanish were a majority and, therefore, exerted greater influence. Moreover, there was no representation from other important countries such as Colombia, Cuba, Peru and Venezuela). The technique used in the study for selection of the experts (snowball sample) allows for the identification of a potential expert through an active search through networks of potential experts and a consensus involving multiple recommendations by their peers. This was considered a more comprehensive way of carrying out the selection procedure than when done by direct selection of experts^60^. Therefore, a biased selection (based on the knowledge and convenience of the initial committee members) would have been mitigated^61^. The above arguments make it reasonable to accept validity of the proposal. The extensive literature review included a comprehensive study of the most relevant recommendations and proposals regarding clinical Nursing communication. The preliminary discussion about the theoretical model and its adaptation to the Nursing practice, as well as about the communication domains to be considered here, facilitated a reasoned selection of the preliminary LO list. Despite this, the outcome reveals a perspective of clinical communication and its main elements that can also be criticised from the point of view of other theoretical frameworks^62^. The criterion used to mark consensus has been statistical and, although accepted, is nevertheless a discretionary criterion. Finally, another important aspect that supports the decision to employ a consensus method is that the resulting statement should be adopted and used by as many institutions, boards and organisations as possible. To achieve this goal, it is important to involve stakeholders in the development of the consensus statement and not in the implementation process alone^63^. Due to the risks involved in unstructured debates, we believe it difficult to imagine a better way to standardise and ensure the process and its ensuing results.

Bearing all this in mind, the consensus method employed may be considered as one of the main strengths of this proposal, as it reflects the communicative requirements of a Nursing practice that can be taught to students. Finally, it can be observed that, although the experts came from countries that share the same language, there exist notable socio-cultural and economic differences between some of them. Even so, a possible biased preliminary choice of items by the Scientific Committee by the predominant cultural subgroup within the main group cannot be ruled out.

## Conclusion

The current proposal of 68 LOs in nurse-patient communication for Nursing degree studies reached by consensus among 70 Spanish-speaking participants of different backgrounds and professional profiles may help design and incorporate communication programs for Nursing students, depending on the priorities and circumstances that each institution concerned may consider to be more appropriate for its graduates. This proposal may also serve as a useful guide for the development of didactic strategies and the evaluation of this type of communication skills. Finally, the broad consensus reached among a high number of experts in diverse areas of the health care professions make CHCE a tool that helps increase awareness and dissemination of educational programs of nurse-patient communication in Nursing schools among the countries concerned and also in other countries with similar characteristics.
